# Anemia in a young Guinean male

**DOI:** 10.1002/ccr3.4593

**Published:** 2021-08-06

**Authors:** Nicholas S. Wilcox, Chantal Tapé, Anthony J. Cordisco, Minh T. Than, Leah Zuroff, Jane Dobkin, Ryan C. Massa, Andrew G. Lytle, Adam Bagg, Htun Min, Laurel J. Glaser, Laura M. Kosseim

**Affiliations:** ^1^ Department of Medicine Perelman School of Medicine University of Pennsylvania Philadelphia PA USA; ^2^ Department of Pathology Perelman School of Medicine University of Pennsylvania Philadelphia PA USA

**Keywords:** anemia, hematology, infectious diseases, pernicious anemia, schistosomiasis, vitamin B12 deficiency

## Abstract

The etiology of anemia in adults is often multifactorial. This case highlights an uncommon combination of causes of anemia and the importance of a diagnostic workup guided by a patient's unique history, risk factors, and laboratory findings.

## Graphical abstract

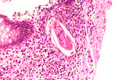

## 1 INTRODUCTION

Anemia is the most common hematologic disorder and is often multifactorial in etiology. In a 23‐year‐old Guinean male, we identify the first known case of anemia due to schistosomiasis and pernicious anemia. The patient ultimately improved with a combination of supportive care, Praziquantel, and vitamin B12 supplementation.

Anemia, defined as a reduction in the absolute number of circulating red blood cells, is the most common hematologic disorder, affecting almost 1/4 of the world's population.^1^ Anemia is rarely a normal finding and may reflect one or more underlying disease processes. Broadly, this includes bleeding, increased destruction or underproduction of red blood cells. In adult patients, the etiology of anemia may be typically multifactorial. Characteristic signs and symptoms result from reduced oxygen delivery to tissues and, in the case of bleeding, hypovolemia. Here, we present a clinical case of an otherwise healthy male of Guinean descent with severe, symptomatic anemia secondary to two uncommon processes: pernicious anemia and schistosomiasis resulting in lower gastrointestinal bleeding. The findings in this case underscore the importance of pursuing a thorough diagnostic workup informed by the patient's unique history, physical exam, and initial laboratory findings.

## 2 CASE HISTORY/EXAMINATION

The patient, a 23‐year‐old male, initially presented to his primary care physician reporting 3 weeks of intermittent bright red blood per rectum with occasional melanotic stools in the setting of 2 months of generalized fatigue. He also reported postprandial abdominal pain with associated intermittent non‐bilious and non‐bloody vomiting and a 15‐pound unintentional weight loss since symptom onset. This occurred in the context of a 2‐year history of occasional, small‐volume bloody stools, which had been previously been attributed to hemorrhoids. Outpatient complete blood count (CBC) revealed a hemoglobin of 4.5 g/dL, for which he was advised to present to the Emergency Department (ED).

There, the patient appeared tired but was in no acute distress. He was breathing comfortably on room air and fully alert and oriented. He denied neurologic, infectious, cardiopulmonary, genitourinary or dermatologic signs, and symptoms. His past medical history was notable only for hemorrhoids and his only prior surgery was orchiopexy in childhood. The patient denied recent sick contacts, travel or taking medications. Social history was pertinent for the patient immigrating from Guinea to New York City at the age of eleven, before relocating to Philadelphia one year prior to presentation. He notably lacked health insurance, and this was his first visit to a hospital in the United States. He reported occasional marijuana and social alcohol use but denied use of non‐steroidal anti‐inflammatory drugs (NSAIDs) or illicit drugs. Family history was significant for one grandparent with late onset colon cancer but otherwise unremarkable.

Vitals were significant for sinus tachycardia but otherwise within normal limits. Physical exam was notable for anicteric sclera, moderate conjunctival pallor, a normal cardiopulmonary exam, normoactive bowel sounds, and a non‐tender, non‐distended abdomen without hepatosplenomegaly. Rectal exam revealed light brown, heme‐negative stool in vault. Extremities were warm and well‐perfused without rashes or petechiae.

### 2.1 Differential diagnosis, investigations, and treatment

In the ED, the patient's CBC was notable for a normocytic anemia with a hemoglobin (Hgb) of 4.3 g/dl, mean corpuscular volume (MCV) of 94 fl, and profoundly elevated red cell distribution width (RDW) >40.0%. Notably, the differential did not show peripheral eosinophilia. The patient was also thrombocytopenic with platelets of 80,000/μl. Leukocyte count was initially normal, but he was transiently leukopenic on hospital days 3 and 4, with a nadir of 3400/μl. The patient received 3 units of packed red blood cells (pRBCs) with an appropriate increase in his Hgb to 8.1 g/dl.

**FIGURE 1:**
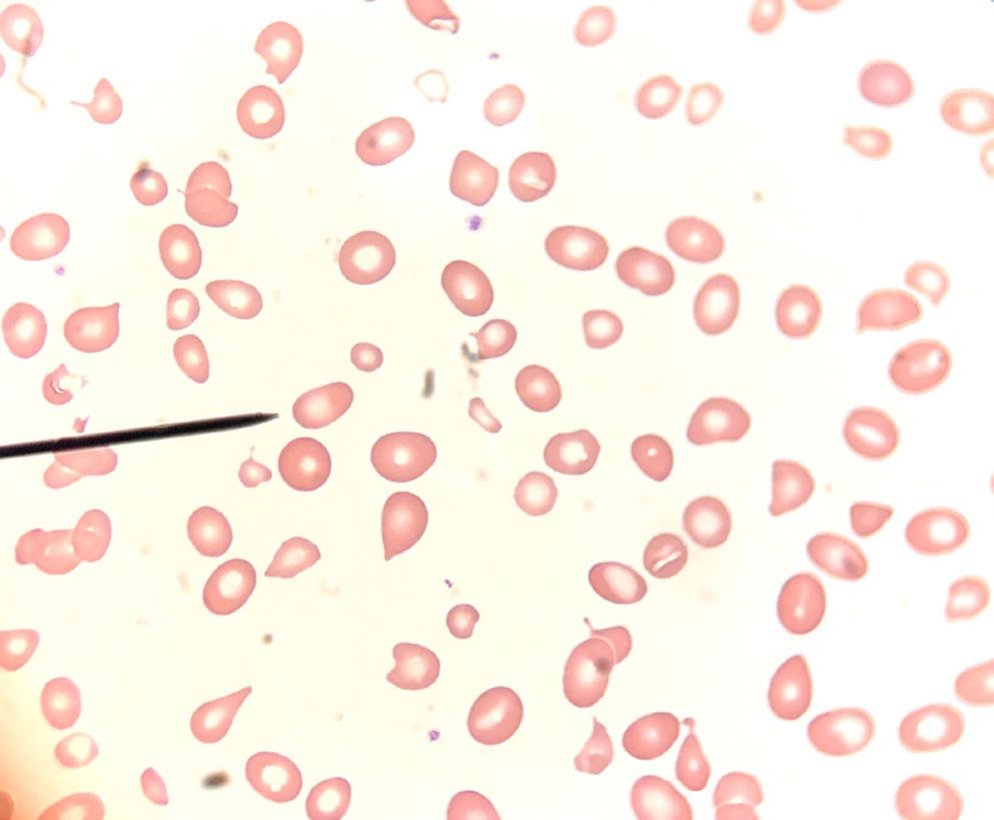
Peripheral blood smear. Peripheral blood smear notable for anisopoikilocytosis, numerous tear drop cells, rare schistocytes, without a leukoerythroblastic picture

Further laboratory studies demonstrated an elevated lactate dehydrogenase (LDH) of 6913 U/L, undetectable haptoglobin, and increased total and indirect bilirubin (1.6 and 1.2 mg/dl, respectively), consistent with hemolysis. Liver function tests were notable for mild transaminitis, with alanine transaminase (ALT) of 66 U/L and aspartate transaminase (AST) of 192 U/L alkaline phosphatase, however, was normal. Peripheral blood smear showed marked red blood cell anisocytosis, with rare schistocytes, ovalocytes, and numerous teardrop cells noted (Figure 1). No parasites were visualized in the peripheral blood.

Direct Coombs’ testing was negative, ruling out autoimmune hemolysis. Fibrinogen and coagulation studies including PT/PTT/INR were normal, which along with the only rare schistocytes on peripheral smear made a thrombotic microangiopathy less likely. Erythrocyte G6PD activity assay was not performed as this may be falsely normal in G6PD‐deficient patients in the setting of active hemolysis. Various microbes can cause hemolysis and/or cytopenias, but human immunodeficiency virus (HIV) IgG, rapid plasma reagin (RPR), Interferon gamma (IFN)‐γ release assay, *Ehrlichia* polymerase chain reaction (PCR), *Anaplasma* PCR, and *Babesia* IgG were all negative. Sickle cell preparation and Hgb electrophoresis were negative for underlying hemoglobinopathy. Parvovirus B19 can cause red cell aplasia even without sickle cell disease/trait but PCR was negative for active infection.

The striking anisocytosis suggested multiple pathologic processes producing cell populations of different sizes. The absolute reticulocyte count was 36.0 (2.7%), suggesting an inappropriately hypoproliferative erythropoietic response to anemia. Iron and transferrin saturation were slightly elevated (187 μg/dl and 58%, respectively), with normal transferrin and elevated ferritin (411.8 ng/ml), consistent with anemia of chronic inflammation. Serum vitamin B12 levels were decreased (116 pg/ml), suggesting a megaloblastic contribution to the anemia and anisocytosis. Folate was near the lower limit of normal (9 ng/ml). The patient was started on oral vitamin B12 supplementation at a dose of 2000 mcg daily while inpatient.

**FIGURE 2:**
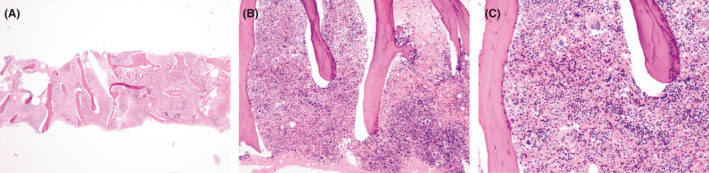
A–C: Pathology from core biopsy of bone marrow. Hematoxylin and eosin stain staining of the bone marrow core biopsy showing hypercellular marrow (95%) with erythroid and megakaryocytic hyperplasia with mildly left‐shifted granulopoiesis. There neither an abnormal infiltrate nor increased fibrosis

Though teardrop cells can be seen in vitamin B12 deficiency, iron deficiency, and thalassemia, they are classically associated with anemias due to bone marrow infiltration, or myelophthisis.^2^ Such processes include myelofibrosis (primary or arising from myelodysplasia), malignancy (hematologic or metastatic carcinomas of the breast, prostate, etc.), lipid storage diseases, and granulomatous inflammation, including miliary tuberculosis and sarcoidosis.^3^ In this patient, the numerous teardrop cells appreciated on peripheral smear in the setting of a hypoproliferative reticulocyte count warranted a bone marrow biopsy to rule out myelophthisic anemia. Pathology showed a hypercellular (95%) marrow with erythroid and megakaryocytic hyperplasia, normal myeloid and erythroid development, normal lymphocyte number and morphology, and no fibrosis or metastatic disease (Figure 2). While non‐specific, these findings were most consistent with the vitamin B12 deficiency, albeit with the absence of prototypic megaloblastic morphology due to the coexistent iron‐deficient hematopoiesis.

**FIGURE 3:**
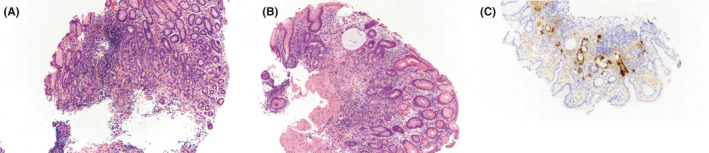
A–C: Pathology from biopsy of stomach. Fragments of benign gastric mucosa showing chronic inflammation and focal intestinal metaplasia. Chromogranin immunostain reveals nodular endochromaffin cell hyperplasia consistent with chronic atrophic gastritis

**FIGURE 4:**
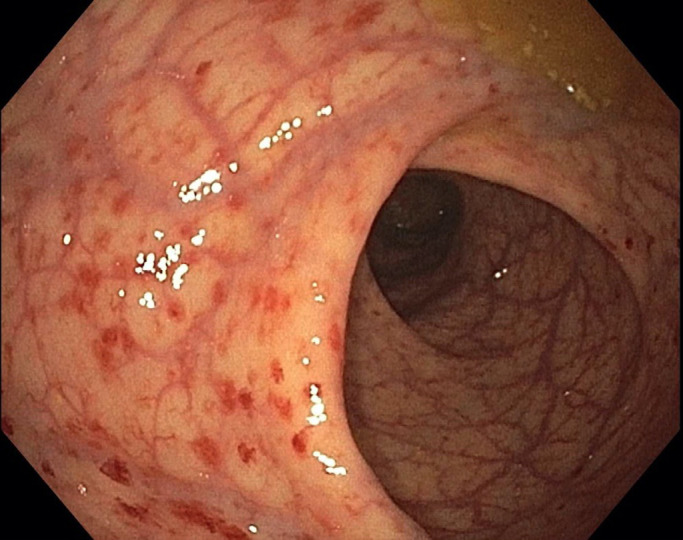
Colonoscopy images of distal rectum. Colonoscopy showed normal perianal and digital rectal examinations were normal. There was patchy moderate erythema in the distal rectum. The terminal ileum and the rest of the colon appeared normal

**FIGURE 5:**
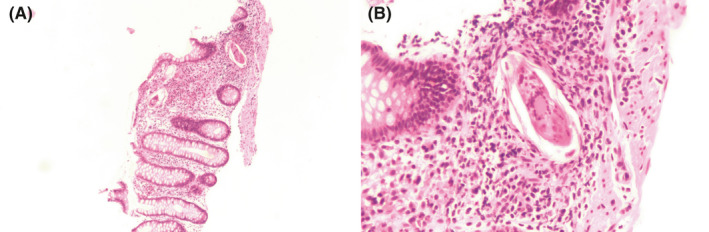
A and B: Pathology from biopsy of colonic rectum. Schistosoma forms within colonic mucosa with mild acute inflammation and focal presence of multinucleated giant cells

Upper endoscopy and colonoscopy were also performed to evaluate the patient's history of gastrointestinal bleeding. Biopsies of the gastric mucosa demonstrated chronic inflammation, intestinal metaplasia, and endochromaffin cell hyperplasia consistent with atrophic gastritis (Figure 3). *H*. *pylori* immunohistochemistry was negative. Anti‐parietal cell antibodies were also negative but anti‐intrinsic factor antibodies returned positive. In light of the patient's low vitamin B12 and atrophic gastritis, this was most consistent with pernicious anemia. During the colonoscopy, direct visual inspection revealed grossly normal enteric mucosa except in the rectum, where there was patchy erythema (Figure 4). Histopathology from rectal biopsy demonstrated schistosoma forms with multinucleated giant cell inflammatory reactions (Figure 5). As such, the patient's bleeding was attributed to chronic intestinal schistosomiasis. Of note, while stool microscopy is generally considered the gold standard for diagnosing schistosomiasis, rectal biopsies actually have higher diagnostic sensitivity.^4^ Rectal biopsies are particularly useful when there is a high clinical suspicion for schistosomiasis despite negative laboratory‐based testing.

Praziquantel is the treatment of choice for schistosomiasis.^5^ Stool ova and parasite assay was performed to identify the *Schistosoma* species involved as the ideal dosing is somewhat species‐dependent.^5^ However, no ova or parasites were visualized. Therefore, the patient was given a dose of 40 mg/kg praziquantel empirically as this dose is appropriate for *S*. *mansoni* and *S*. *hematobium*, both of which are endemic to Guinea.^5^ The patient tolerated this treatment without appreciable side effects.

### 2.2 Outcome and follow‐up

At the time of outpatient follow‐up with infectious disease approximately 3 weeks post‐discharge, the patient's symptoms of fatigue and hematochezia had resolved, and he had regained the 15 pounds of unintended weight loss. That week, the patient received his second and final dose of Praziquantel without complication. His vitamin B12 level at the time of initial follow‐up with hematology was normal (643 pg/ml) and repeat CBC showed a now mild normocytic anemia (Hgb 12.8, MCV 88 fl) with improved RDW of 14.3%. As such, the patient was advised to reduce the dose of supplemental oral Vitamin B12 to 1000 mcg daily. Repeat studies were consistent with iron deficiency anemia (Ferritin 11.3 ng/ml), which had been previously masked by anemia of chronic inflammation, suggesting mobilization of iron stores accompanying the patient's improved hemoglobin. For this, the patient was started on oral ferrous sulfate 325 mg twice daily. Given the patient's autoimmune atrophic gastritis, a repeat outpatient endoscopy was scheduled with gastroenterology for 3 months post‐discharge for mapping and risk stratification for gastric cancer.

## 3 DISCUSSION

The natural history and spectrum of tissue involvement of schistosomiasis infection enables this parasite to induce a variegated array of clinical symptoms. Acute infections in naïve hosts result in localized dermatitis at the site of cercarial penetration, and subsequent dissemination, maturation of schistosomes, and egg‐laying to trigger Acute Schistosomiasis (AS). However, in hosts living within endemic regions, AS is rare, presumably through in utero desensitization.^2^ Instead, these hosts have a prolonged infectious course as egg antigens within tissue parenchyma provoke granuloma formation. Classically, intestinal, hepatic, pulmonary, urinary, genital, and neurological functions can be compromised with established and chronic infection.

Our patient demonstrated a constellation of symptoms consistent with late chronic schistosomiasis infection. Notably, he emigrated from an endemic area of West Africa and presented with symptomatic anemia, months of vague GI symptoms, and intermittent episodes of melena and hematochezia. Intestinal schistosomiasis is thought to cause iron deficiency anemia through GI blood loss. Thus, these symptoms could be explained alone by intestinal schistosomiasis, which was ultimately confirmed by colonoscopy and subsequent biopsy.

However, further workup revealed multifactorial causes of this patient's anemia, which are not previously described in canonical cases of chronic schistosomiasis infection. Of note, he showed evidence of hemolysis without autoantibodies, pernicious anemia with atrophic gastritis, and a blood smear with significant tear drop cells. Mechanistic investigations have previously demonstrated that irradiated cercariae can result in an autoimmune hemolytic anemia.^3^ Our patient's vitamin B12 deficiency contributed to ineffective erythropoiesis and associated intramedullary destruction of RBC precursors, which in turn led to laboratory findings consistent with a hemolytic process including elevated LDH levels.

Micronutrient deficiency, including B12 and folate deficiency as observed in our patient, has been observed in chronic schistosomiasis, presumably from malabsorption and reduced dietary intake due to poor appetite.^4^, ^5^ Presence of dacrocytes is usually suggestive of a myelophthisic process, which is rare but has previously been identified in the bone marrow of a patient with schistosomiasis.^6^ However, our patient's bone marrow pathology was non‐specific and did not demonstrate findings characteristic of a myelophthisic process including fibrosis or infiltrate. Finally, our patient uniquely had both schistosomiasis and pernicious anemia, and not solely B12 deficiency due to malabsorption, in contrast to prior cases.

The immune response to schistosomes drives the underlying symptomatology of this disease.^7^ As such, we postulate that the collection of these atypical features are further extensions of chronic schistosomiasis. How vitamin B12 deficiency and pernicious anemia are mechanistically related to the pathogenesis and immune response to schistosomiasis is deserving of further investigation, given that regions with the highest prevalence of B12 deficiency also bear the greatest burden of neglected tropical diseases.^8^ This case highlights the importance of thorough diagnostic evaluation of anemia as well as emphasizes unique manifestations of schistosomiasis.

## CONFLICTS OF INTEREST

The authors have no funding sources or conflicts of interest to disclose.

## AUTHOR CONTRIBUTIONS

NSW, CT, AJC, MTT, LZ and JD played a substantial role in data analysis, interpretation, manuscript preparations and revisions. RCM, AGL, AB, HM, LJG and LMK substantially contributed to data analysis, interpretation and manuscript revisions. All authors have given final approval for the manuscript to be published and are accountable for the accuracy and integrity of the paper.

## ETHICAL STATEMENT

Ethics approval was not required for this study.

## CONSENT STATEMENT

All the listed authors consent for publication.

## PATIENT CONSENT

The patient provided written consent ahead of submission.

## Data Availability

The data that support the findings of this study are available on request from the corresponding author. The data are not publicly available due to privacy or ethical restrictions.
